# Riboflavin/UVA Collagen Cross-Linking-Induced Changes in Normal and Keratoconus Corneal Stroma

**DOI:** 10.1371/journal.pone.0022405

**Published:** 2011-08-05

**Authors:** Sally Hayes, Craig Boote, Christina S. Kamma-Lorger, Madhavan S. Rajan, Jonathan Harris, Erin Dooley, Nicholas Hawksworth, Jennifer Hiller, Nick J. Terill, Farhad Hafezi, Arun K. Brahma, Andrew J. Quantock, Keith M. Meek

**Affiliations:** 1 Structural Biophysics Research Group, School of Optometry and Vision Sciences, Cardiff University, Cardiff, United Kingdom; 2 Vision and Eye Research Unit, Anglia Ruskin University, Cambridge, United Kingdom; 3 Royal Glamorgan Hospital, Llantrisant, Rhondda Cynon Taff, United Kingdom; 4 Diamond Light Source, Didcot, Oxfordshire, United Kingdom; 5 Division of Ophthalmology, Geneva University Hospitals, Geneva, Switzerland; 6 Manchester Royal Eye Hospital, Manchester, United Kingdom; University of Reading, United Kingdom

## Abstract

**Purpose:**

To determine the effect of Ultraviolet-A collagen cross-linking with hypo-osmolar and iso-osmolar riboflavin solutions on stromal collagen ultrastructure in normal and keratoconus *ex vivo* human corneas.

**Methods:**

Using small-angle X-ray scattering, measurements of collagen D-periodicity, fibril diameter and interfibrillar spacing were made at 1 mm intervals across six normal post-mortem corneas (two above physiological hydration (swollen) and four below (unswollen)) and two post-transplant keratoconus corneal buttons (one swollen; one unswollen), before and after hypo-osmolar cross-linking. The same parameters were measured in three other unswollen normal corneas before and after iso-osmolar cross-linking and in three pairs of swollen normal corneas, in which only the left was cross-linked (with iso-osmolar riboflavin).

**Results:**

Hypo-osmolar cross-linking resulted in an increase in corneal hydration in all corneas. In the keratoconus corneas and unswollen normal corneas, this was accompanied by an increase in collagen interfibrillar spacing (p<0.001); an increase in fibril diameter was also seen in two out of four unswollen normal corneas and one unswollen keratoconus cornea (p<0.001). Iso-osmolar cross-linking resulted in a decrease in tissue hydration in the swollen normal corneas only. Although there was no consistent treatment-induced change in hydration in the unswollen normal samples, iso-osmolar cross-linking of these corneas did result in a compaction of collagen fibrils and a reduced fibril diameter (p<0.001); these changes were not seen in the swollen normal corneas. Collagen D-periodicity was not affected by either treatment.

**Conclusion:**

The observed structural changes following Ultraviolet-A cross-linking with hypo-osmolar or iso-osmolar riboflavin solutions are more likely a consequence of treatment-induced changes in tissue hydration rather than cross-linking.

## Introduction

Corneal collagen cross-linking therapy is a technique that uses a combination of riboflavin (vitamin B_2_) and ultraviolet-A light (UVA) to induce cross-linking in stromal collagen and thereby treat corneal ectasia occurring in keratoconus [1], [2], [3] or following laser refractive surgery [4], [5], [6]. It is thought to work by enhancing the biomechanical properties of the tissue [7], [8] and its resistance to enzymatic digestion [9]. The photosensitiser riboflavin is applied to the de-epithelialised surface of the cornea and allowed to penetrate into the corneal stroma [10], [11]. The subsequent exposure of the cornea to UVA light is thought to result in photodynamic cross-linking when the riboflavin, excited by UVA, creates free radicals leading to cross-linking of collagen [11]. Until recently, this treatment was deemed unsuitable for corneas with a stromal thickness of less than 400 µm due to the potential for damage to the endothelium and deeper ocular structures [11]. However, a recent modification to the technique in which the standard iso-osmolar riboflavin solution (containing dextran) is substituted with a hypo-osmolar riboflavin solution (without dextran) to induce stromal swelling and increase stromal thickness prior to cross-linking, has enabled the treatment to be performed on very thin keratoconus corneas (<400 µm) that would not have previously been eligible for riboflavin/UVA treatment [12].

Despite the increasing popularity of the approach, little is known about the specific nature of the cross-links that are formed as a result of riboflavin/UVA cross-linking, or about their location either within collagen fibrils or in the interfibrillar matrix. In principle, many of the results obtained to date could be explained by assuming cross-links occur at the surface of the collagen fibrils or between the fibrils and the proteoglycan-rich extrafibrillar matrix. Small-angle X-ray scattering is a non-invasive technique that provides information about the axial structure and diameter of collagen fibrils, as well as the distance by which neighbouring fibrils are separated from each other within the corneal stroma [13]. The data obtained are averages from every collagen fibril in the thickness of the cornea through which the X-rays pass, and are thus highly representative of the tissue as a whole.

In this study small-angle X-ray scattering was employed to examine the structural changes that occur within the stroma of normal and keratoconus corneas following UVA collagen cross-linking therapy using hypo-osmolar and iso-osmolar riboflavin solutions.

## Methods

### Ethics statement

The research presented in this manuscript was approved by the Human Science Ethical Committee (School of Optometry and Vision Sciences, Cardiff University, UK) and the South East Wales Research Ethics Committee (Cardiff, UK). The institutional review board approved the use of all corneas described in this study; a waiver of consent was given for the fifteen donor cormeas, as these were obtained from the Bristol eye bank. All tissue used in this study was obtained in accordance with the tenets of the Declaration of Helsinki, and local ethical rules were adhered to throughout.

### Tissue

Two keratoconus corneal buttons (one from a patient aged 24; one unknown) were obtained with written patient consent from the Royal Glamorgan Eye Hospital (Llantrisant, UK) following penetrating keratoplasty. Cornea K1 (8 mm button) had a stellate scar in the anterior stroma and a scar across the centre of the cornea at the level of Descemet's membrane. Cornea K2 (7.5 mm button) showed slight superficial scarring. Both corneas were less than 400 µm thick.

In addition, fifteen normal donor corneas, ranging from 61 to 87 years in age, were obtained from the Corneal Transplant Service (Bristol Eye Bank, UK). Nine of the corneas (N1–9) were single specimens from individual donors but six of the corneas (N10+N11, N12+N13, N14+N15) were left/right pairs from the same donor. The normal corneas, which were deemed unsuitable for transplantation surgery due to a low endothelial cell count, had been stored in culture medium (minimum essential medium+2% fetal calf serum) for several weeks and thus presented a range of above physiological tissue hydrations. On receipt, both keratoconus corneal buttons (K1–2) and two of the normal corneas (N5–6) were wrapped tightly in Clingfilm™ (Superdrug Stores, Croydon, UK) to prevent tissue dehydration and stored at −80°C until required for experimentation. The remaining normal corneas (N1–4 and N7–N15) were not frozen at any stage.

Prior to data collection, the frozen corneas (K1–2 and N5–6) were thawed at room temperature. Seven of the normal corneas (N1–4 and N7–9) were air dried to near physiological hydration prior to data collection and cross-linking. During the air drying process, pachymetry was used to monitor corneal thickness every 5 minutes; air drying was terminated when a tissue thickness of 500–550 microns was reached and the tissue deemed to be ‘unswollen’. The cornea was then weighed and wrapped tightly in clingfilm to prevent further drying during data collection. The remaining normal corneas (N5–6 and N10–N15) were examined using X-rays and, in the case of N5–N6, N10, N12 and N14 were also cross-linked, whilst they were still swollen.

Due to the sensitivity of collagen interfibrillar spacing to changes in corneal hydration [14] it was necessary to calculate tissue hydration pre and post cross-linking. This was done using the equation: Hydration = (wet weight-dry weight)/dry weight. As the pre-treatment hydration varied between corneas at the start of the study (H = 1.9 to 6.6), owing to conditions of storage and handling, they have been classified as being swollen (above a physiological hydration of H = 3.2) or unswollen (at or below H = 3.2). Sample details and classification have been summarised in Table 1.

**Table 1:** Sample details and treatments.

Sample	Hydration	Classification	Riboflavin/UVA treatment
**Hypo-osmolar riboflavin/UVA cross-linking: Keratoconus**			
K1	2.2	Keratoconus; unswollen	Hypo-osmolar
K2	3.8	Keratoconus; swollen	Hypo-osmolar
**Hypo-osmolar riboflavin/UVA cross-linking: Normal**			
N1	2.3	Normal; unswollen	Hypo-osmolar
N2	2.4	Normal; unswollen	Hypo-osmolar
N3	2.1	Normal; unswollen	Hypo-osmolar
N4	2.5	Normal; unswollen	Hypo-osmolar
N5	6.6	Normal; swollen	Hypo-osmolar
N6	6.6	Normal; swollen	Hypo-osmolar
**Iso-osmolar riboflavin/UVA cross-linking: Normal**			
N7	2.0	Normal; unswollen	Iso-osmolar
N8	1.9	Normal; unswollen	Iso-osmolar
N9	2.5	Normal; unswollen	Iso-osmolar
N10	5.7*	Normal left; swollen	Iso-osmolar
N11	5.7	Normal right; swollen	None (control for N10)
N12	5.3*	Normal left; swollen	Iso-osmolar
N13	5.3	Normal right; swollen	None (control for N12)
N14	5.2*	Normal left; swollen	Iso-osmolar
N15	5.2	Normal right; swollen	None (control for N14)

**Details of pre-treatment tissue hydration (recorded at the time of data collection), classification and UVA cross-linking treatment (hypo-osmolar/iso-osmolar riboflavin solution) are shown for each sample. Each cornea has been classified as being either ‘unswollen’ (at or below a physiological hydration of H = 3.2) or ‘swollen’ (above physiological hydration).**

*The pre-treatment hydration of samples N10, N12 and N14 was assumed to be the same as that of their untreated pair (N11, N13 and N15 respectively).

### Iso-osmolar/Hypo-osmolar riboflavin/UVA cross-linking

The iso-osmolar cross-linking treatment used throughout this study involved the removal of the corneal epithelium followed by a single application of iso-osmolar riboflavin eye drops (containing riboflavin 0.136% and dextran 20%). After five minutes, the cornea was then exposed to a 30 minute 3.04 mW/cm^2^ dose of UVA, during which time the iso-osmolar riboflavin eye drops were re-applied at 3 minute intervals. In the case of hypo-osmolar riboflavin cross-linking, the iso-osmolar riboflavin eye drops were replaced with hypo-osmolar eye drops (containing riboflavin 0.136%, but no dextran).

### Small-angle X-ray scattering data collection and analysis

The corneas (wrapped in Clingfilm) were mounted in a sealed polymethyl methacrylate (Perspex; theplasticshop.co.uk, Coventry, UK) chamber with polyester film (Mylar; DuPont-Teijin, Middlesbrough, UK) windows and positioned ready for X-ray scattering data collection on Station I22 at the Diamond Light Source (Didcot, UK).

Small-angle X-ray scattering patterns were obtained at 1 mm intervals (in a grid) over two keratoconus corneal buttons (K1 and K2), four unswollen normal corneas (N1–4) and two swollen normal corneas (N5 and N6). The corneas were then cross-linked using UVA and a hypo-osmolar riboflavin solution and X-ray scatter patterns again collected at 1 mm intervals over each sample. In the same way, X-ray scatter patterns were obtained from three normal unswollen corneas (N7–N9) before and after iso-osmolar cross-linking. Each X-ray scatter pattern resulted from a 10 s exposure to a 0.1 nm wavelength X-ray beam focussed to measure 0.2×0.2 mm at the specimen. The data were recorded on a detector positioned 5 m (N1–N4 and N7–N9) or 6 m (N5, N6, K1 and K2) behind the specimen. Also examined were three pairs of swollen corneas (N10+N11, N12+N13, N14+N15) in which the left of each pair was cross-linked with iso-osmolar riboflavin solution and UVA and the right served as an untreated control. A single X-ray scatter pattern was collected from the centre of each treated and untreated cornea using an X-ray beam focussed to measure 0.5 mm vertically by 2 mm horizontally.

Each X-ray scatter pattern consisted of an intense equatorial reflection, arising from the regular short-range spacing of collagen fibrils, a fainter and broader subsidiary equatorial maxima, caused by the uniformity of fibril diameters, and a series of sharp meridional reflections arising from the axial periodicity (D-period) along the fibrils. The X-ray scatter patterns were calibrated against the 67 nm meridional spacing of collagen in hydrated rat tail tendon.

Measurements of collagen D-period, fibril diameter and inter-fibrillar Bragg spacing before and after treatment at each measured position in the cornea were determined from the calibrated positions of the meridional [13], subsidiary equatorial maxima [13],[15],[16] and innermost equatorial reflection [13], [16], respectively. The relationship between X-ray Bragg spacing and the corresponding centre-to-centre distance of the parameter under investigation depends on the precise packing of the fibrils within the stroma. Most previous investigations have assumed a liquid-like or quasi-hexagonal packing [15], [17], in which case Bragg spacings need to be multiplied by a factor of 1.12 in order to convert to centre-to-centre spacings. However, as we are only concerned here with changes in these parameters, we present all results as Bragg spacings.

A paired student t-test was used to statistically examine differences between cross-linked and non-crosslinked specimens in terms of collagen fibril diameter, interfibrillar spacing and D-periodicity. Based on the accuracy with which measurements of the various collagen parameters could be made, a probability of P<0.001 was taken to indicate a statistically significant treatment difference.

## Results

Table 2 summarises the changes in hydration, collagen fibril diameter, interfibrillar spacing and D-periodicity in normal and keratoconus corneas following iso-osmolar and hypo-osmolar riboflavin/UVA collagen cross-linking. The contents of the table are discussed below.

**Table 2:** Tissue hydration and collagen parameters measured before (CXL-) and after (CXL+) riboflavin/UVA collagen cross-linking.

Sample	Hydration		Fibril Diameter (nm)		Interfibrillar Spacing (nm)		D-period (nm)	
	CXL−	CXL+	CXL−	CXL+	CXL−	CXL+	CXL−	CXL+
**Hypo-osmolar riboflavin/UVA cross-linking: Keratoconus**								
K1	2.2	4.0	**31.1 (0.1)**	**32.8** **(0.1)**	**38.3 (0.3)**	**47.3 (0.2)**	65.4 (0.1)	65.5 (0.1)
K2	3.8	6.0	31.6 (0.1)	32.0 (0.2)	**49.7 (0.3)**	**61.2 (0.4)**	65.5 (0.1)	65.4 (0.1)
**Hypo-osmolar riboflavin/UVA cross-linking: Normal**								
N1	2.3	3.5	**34.5 (0.1)**	**34.8 (0.1)**	**48.1 (0.1)**	**51.1 (0.2)**	65.2 (0.1)	65.2 (0.1)
N2	2.4	4.1	32.9 (0.3)	32.7 (0.2)	**48.2 (0.2)**	**52.6 (0.2)**	66.1 (0.1)	66.1 (0.1)
N3	2.1	2.9	34.3 (0.1)	34.6 (0.1)	**50.5 (0.1)**	**52.6 (0.2)**	65.2 (0.1)	65.2 (0.1)
N4	2.5	3.4	**33.4 (0.1)**	**33.8 (0.1)**	**50.7 (0.3)**	**54.2 (0.3)**	66.1 (0.1)	66.1 (0.1)
N5	6.6	6.9	33.8 (0.2)	33.5 (0.1)	58.6 (0.4)	60.2 (0.3)	64.7 (0.1)	64.5 (0.1)
N6	6.6	6.8	33.2 (0.2)	33.8 (0.4)	57.9 (0.3)	57.2 (0.3)	63.7 (0.1)	63.5 (0.1)
**Iso-osmolar riboflavin/UVA cross-linking: Normal**								
N7	2.0	2.2	**33.6 (0.1)**	**31.0 (0.1)**	**50.0 (0.1)**	**39.2 (0.1)**	66.1 (0.1)	66.1 (0.1)
N8	1.9	1.8	**33.5 (0.1)**	**32.4 (0.1)**	**49.2 (0.3)**	**44.9 (0.3)**	65.2 (0.1)	65.2 (0.1)
N9	2.5	2.7	**34.0 (0.1)**	**31.9 (0.1)**	**49.1 (0.2)**	**41.6 (0.3)**	65.2 (0.1)	65.2 (0.1)
N10	-	5.4	-	33.1	-	46.5	-	66.0
N11	5.7	-	33.7	-	49.1	-	66.0	-
N12	-	5.1	-	33.3	-	48.5	-	66.0
N13	5.3	-	32.1	-	49.1	-	66.0	-
N14	-	4.7	-	33.3	-	50.4	-	66.0
N15	5.2	-	33.7	-	51.0	-	66.0	-

**Average values (+/− SEM) of collagen parameters for all samples (excluding N10–N15) were calculated using >50 measurements recorded from the central 8 mm region of the same corneas before (CXL−) and after cross-linking (CXL+). Averaged data shown for samples N10–N15 (3 pairs of corneas in which the left of each pair (N10, N12 and N14) was cross-linked (CXL+) and the right (N11, N13 and N15) remained untreated (CXL−)) is based on a single measurements obtained from the centre of each cornea. Bold type is used to indicate pre and post treatment differences in collagen parameters at p<0.001.**

### Hypo-osmolar riboflavin/UVA cross-linking of keratoconus corneas

**Figure 1:**
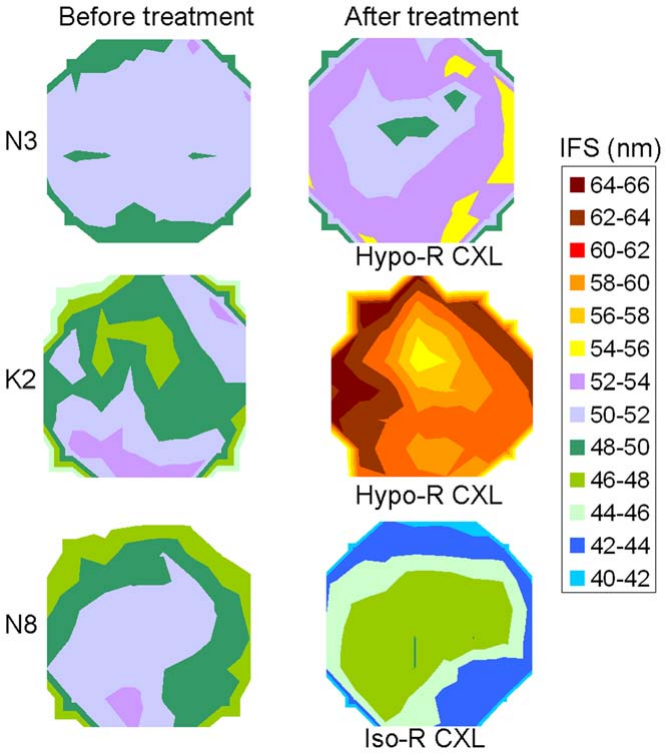
Changes in collagen interfibrillar spacing following Riboflavin/UVA cross-linking. The contour maps show collagen interfibrillar spacing (IFS) in an unswollen normal cornea (N3) and a slightly swollen keratoconus cornea (K2) before and after hypo-osmolar riboflavin/UVA cross-linking (Hypo-R CXL) and a normal unswollen cornea (N8) before and after iso-osmolar riboflavin/UVA cross-linking (Iso-R CXL). The centre of each corneal button corresponds approximately with the centre of each map.

Tissue hydration increased in both keratoconus corneas following hypo-osmolar riboflavin/UVA cross-linking. In each case, this was accompanied by an increase in collagen interfibrillar spacing (p<0.001) (Figure 1).

**Figure 2:**
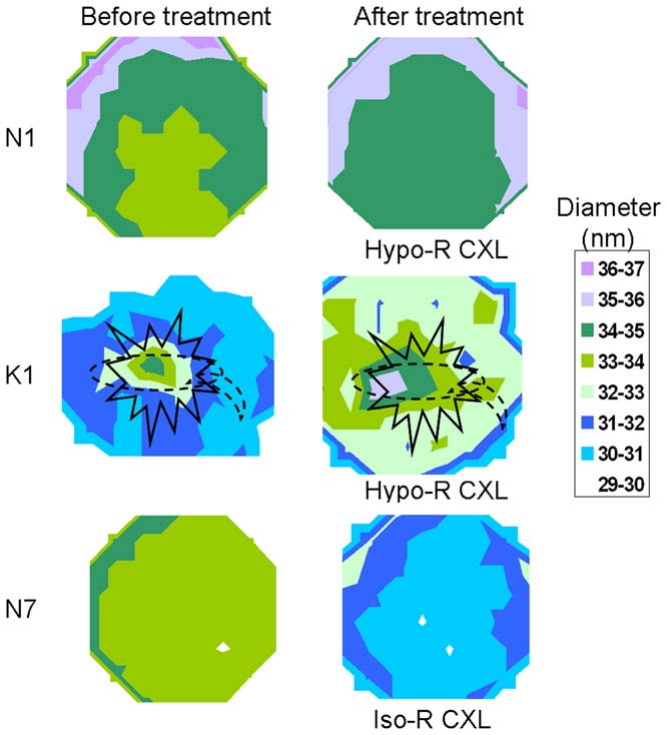
Changes in collagen fibril diameter following Riboflavin/UVA cross-linking. The contour maps show collagen fibril diameter in an unswollen normal (N1) and keratoconus (K1) cornea before and after hypo-osmolar riboflavin/UVA collagen cross-linking (Hypo-R CXL) and an unswollen normal cornea (N7) before and after iso-osmolar riboflavin/UVA collagen cross-linking (Iso-R CXL). The centre of each corneal button corresponds approximately with the centre of each map. The locations of the stellate scar (solid line) and the scar at Descemet's membrane level (broken line) (as recorded schematically by the operating surgeon) have been superimposed onto the contour maps of K1.

In the unswollen cornea (K1), an increase in fibril diameter was observed following hypo-osmolar riboflavin/UVA cross-linking (p<0.001) (Figure 2) but in the swollen cornea (K2) fibril diameter appeared to be unaffected by the same treatment. Interestingly, the region of highest pre-treatment fibril diameter in sample K1 coincided with the site of the stellate scar in the anterior stroma and the scar across the centre of the cornea at the level of Descemet's membrane (as recorded schematically by the operating surgeon prior to performing penetrating keratoplasty). The scarred region also showed the greatest percentage change (12–14% increase) in fibril diameter following treatment (Figure 2).

The D-periodicity of corneal collagen in keratoconus corneas did not change following hypo- osmolar riboflavin/UVA collagen cross-linking treatment.

### Hypo-osmolar riboflavin/UVA cross-linking of normal corneas

Hypo-osmolar riboflavin/UVA cross-linking resulted in an increased tissue hydration in all normal corneas, and a significant increase in collagen interfibrillar spacing in the unswollen corneas (N1–N4) (p<0.001) (Figure 1).

Fibril diameter increased significantly following hypo-osmolar riboflavin/UVA cross-linking treatment in two (N1 and N4) out of four unswollen corneas (p<0.001) (Figure 2). In the remaining unswollen (N2 and N3) and swollen (N5 and N6) corneas, no treatment-induced change in fibril diameter was detected.

Collagen D-periodicity did not change as a result of hypo-osmolar riboflavin/UVA collagen cross-linking treatment in normal corneas.

### Iso-osmolar riboflavin/UVA cross-linking of normal corneas

There was no consistent change in tissue hydration following iso-osmolar riboflavin/UVA cross-linking of unswollen corneas (N7–N9). However, in each pair of swollen corneas, tissue hydration was always lower in the treated cornea (N10, N12, N14) compared to its untreated control (N11, N13, N15).

As shown in Table 1 and Figure 1, iso-osmolar riboflavin/UVA cross-linking resulted in a significant decrease in collagen interfibrillar spacing in the unswollen corneas (N7–N9) (p<0.001). Interfibrillar spacing was also lower in the treated cornea (N10, N12, N14) of each pair of swollen corneas compared to its untreated partner (N11, N13, N15), however this difference was found to be non-significant. The lack of significance in this case may be due to the fact that only one measurement was recorded from the centre of the treated/untreated paired corneas (N10–N15), whereas the statistics for each unswollen cornea (N7–N9) were calculated from a much larger number of data points (>50) recorded before and after treatment.

Iso-osmolar riboflavin/UVA cross-linking caused a significant decrease in fibril diameter in all unswollen corneas (N7–N9) (p<0.001) (Figure 2), but no difference in the average diameter of collagen fibrils was detected between the treated and untreated pairs of swollen corneas (N10–N15).

The D-periodicity of corneal collagen did not change following iso- osmolar riboflavin/UVA collagen cross-linking treatment.

## Discussion

Previous X-ray scattering experiments have shown that as the cornea ages (birth to 90 years), cross-linking leads to a 14% increase in the cross-sectional area associated with each molecule within a fibril [18]. It has also been shown that, *in vitro*, the formation of advanced glycation end-products can cause increases in intermolecular spacing of up to 50% [19], [20]. Measurements of fibril diameter may, therefore, be a useful indicator of the occurrence of collagen cross-linking in a tissue at the molecular level.

In a previous preliminary abstract on porcine corneas cross-linked using iso-osmolar riboflavin solution and UVA [21], we reported that cross-linking increased the intermolecular spacing. However, further investigations revealed that the apparent increase was an artefact, and was not due to the cross-linking (S Hayes, CS Kamma-Lorger, C Boote and KM Meek – unpublished data). In this study, no direct structural changes attributable to cross-linkage were found; fibril diameters were seen to increase only when hypo-osmolar conditions were used and only when the cornea was below physiological hydration prior to treatment. This suggests that increases in fibril diameter under these conditions are due to tissue swelling rather than to molecules being pushed further apart by newly formed inter-molecular cross-links. This finding is in agreement with our recent studies examining the change in collagen inter-molecular spacing of iso-osmolar riboflavin/UVA cross-linked pig corneas during the drying process (from H = 2.7 to H = 0), in which it was observed that the reduction in intermolecular spacing was the same in both treated and untreated corneas at all levels of hydration (unpublished). Since the inter-molecular spacing of corneal collagen is known to increase as the hydration of the tissue increases from dry (H = 0) to physiological (H = 3.2) [14] but remains fairly stable thereafter [14], [22], it may be presumed that in swollen corneas, the absence of any cross-linking-induced changes in fibril diameter is due to the covalent cross-links between adjacent collagen molecules already being at their maximum extension prior to treatment. The fact that the largest diameter increase corresponded to the scarred region of one keratoconus button suggests that the scar tissue might contain collagen fibrils with less naturally occurring cross-links, which are thus able to swell more than the other collagen fibrils in that particular cornea. Indeed, corneal wound healing studies in rabbits have shown the presence of abnormal collagen intermolecular cross-linking in scar tissue even after a year of healing [23]. We therefore were unable to confirm the observations of a previous electron microscopy study which showed evidence of larger than normal fibrils after iso-osmolar cross-linking treatment in both the anterior, and to a lesser extent the posterior, of the rabbit cornea [24]. However, several possible explanations exist for the difference in the outcomes of these studies. The first is that species differences in the response of the cornea to cross-linking may exist due to variations in stromal thickness and hence the proportion of the cornea that is cross-linked; as only the anterior 300 microns of the tissue is cross-linked [25] this represents approximately 75% of the rabbit cornea and 55% of the physiologically hydrated human cornea. The proportion of cross-linked human cornea reduces to 35% when the tissue is swollen, based on a calculated corneal thickness of 851 µm at a hydration of H = 6.6 [26]. Secondly, small changes in fibril diameter which are limited to the anterior cornea may be masked by averaging measurements throughout the full thickness of the tissue as has been done here. Another possibility is that the age of the corneas may affect the efficacy of the cross-linking process. The normal corneas used in this study were from older patients, whose collagen was likely already naturally cross-linked due to ageing; these patients would therefore have had a higher inter-molecular spacing of collagen and a larger fibril diameter than that of younger individuals, [18], [22] even before UVA cross-linking.

The absence of any detectable changes between riboflavin/UVA treated and untreated corneas in terms of the D-period of the collagen, when measured as an average throughout the entire thickness of the tissue, leads us to conclude that UVA/riboflavin induced cross-links do not have any measurable effect on the axial stagger or the tilt of the collagen molecules within the fibrils. This is not wholly surprising because it seems that D-spacing is rather insensitive to cross-linking, as even with strong fixative such as glutaraldehyde it only shrinks by less that 0.8% [27]. Additionally, the data presented here confirms the findings of Daxer et al. [22] which showed that at hydrations above H = 2.5, collagen D-periodicity is independent of changes in tissue hydration.

On average, the interfibrillar spacing decreased after iso-osmolar riboflavin/UVA treatment, due to the presence of dextran which acts as a deturgescent. This finding supports our previous data from pig corneas following similar treatment [21]. As fibrils approach each other, ordering would be expected to increase and this is consistent with the reported increase in transparency of hen corneas following iso-osmolar riboflavin/UVA collagen cross-linking [28]. Two of the normal human corneas incubated with hypo-osmolar riboflavin/UVA were quite swollen before the treatment and therefore showed only a modest increase or no change in the interfibrillar spacing. In the unswollen normal and keratoconus corneas, however, interfibrillar spacing increased significantly after hypo-osmolar riboflavin/UVA treatment.

Hypo-osmolar riboflavin solutions have been suggested as a means of swelling thin keratoconus corneas to allow riboflavin/UVA cross-linking without risking damage to the endothelial cells [12]. Corneal thickness was reported to increase by up to 30% after this treatment and there seemed to be no damage to the endothelium. However, it is well known that when the cornea swells, water can enter different compartments, and these can be between lamellae, into voids or “lakes” within lamellae, between the fibrils within the lamellae or within the fibrils themselves [14], [29], [30], [31]. Here we have shown that, although some fluid enters the fibrils, the majority of the swelling occurs between the fibrils. For example, average interfibrillar spacing increases by up to 30% (Fig. 1, K2) following hypo-osmolar riboflavin swelling, which is similar to the increase in overall tissue thickness. This suggests that swelling is primarily intra-lamellar and occurs between the collagen fibrils. However, this swelling has the effect of thinning the effective thickness of the protective riboflavin film, and it was shown that a hypo-osmolar riboflavin solution film has lower viscocity and a lower absorption coefficient for UVA radiation than iso-osmolar riboflavin, and becomes unstable after only 90 seconds [32], so would need to be used with caution in a clinical setting.
